# Quality Assessment of TikTok as a Source of Information About Mitral Valve Regurgitation in China: Cross-Sectional Study

**DOI:** 10.2196/55403

**Published:** 2024-08-20

**Authors:** Nannan Cui, Yuting Lu, Yelin Cao, Xiaofan Chen, Shuiqiao Fu, Qun Su

**Affiliations:** 1 Department of Surgical Intensive Care Unit, The First Affiliated Hospital Zhejiang University School of Medicine Hangzhou China; 2 Department of Ultrasonography, The First Affiliated Hospital Zhejiang University School of Medicine Hangzhou China; 3 Department of Cardiovascular Surgery, The First Affiliated Hospital Zhejiang University School of Medicine Hangzhou China

**Keywords:** mitral valve regurgitation, video quality, TikTok, Journal of American Medical Association, JAMA, Global Quality Score, GQS, PEMAT- A/V, Spearman correlation analysis, Poisson regression analysis

## Abstract

**Background:**

In China, mitral valve regurgitation (MR) is the most common cardiovascular valve disease. However, patients in China typically experience a high incidence of this condition, coupled with a low level of health knowledge and a relatively low rate of surgical treatment. TikTok hosts a vast amount of content related to diseases and health knowledge, providing viewers with access to relevant information. However, there has been no investigation or evaluation of the quality of videos specifically addressing MR.

**Objective:**

This study aims to assess the quality of videos about MR on TikTok in China.

**Methods:**

A cross-sectional study was conducted on the Chinese version of TikTok on September 9, 2023. The top 100 videos on MR were included and evaluated using quantitative scoring tools such as the modified DISCERN (mDISCERN), the Journal of the American Medical Association (JAMA) benchmark criteria, the Global Quality Score (GQS), and the Patient Education Materials Assessment Tool for Audio-Visual Content (PEMAT-A/V). Correlation and stepwise regression analyses were performed to examine the relationships between video quality and various characteristics.

**Results:**

We obtained 88 valid video files, of which most (n=81, 92%) were uploaded by certified physicians, primarily cardiac surgeons, and cardiologists. News agencies/organizations and physicians had higher GQS scores compared with individuals (news agencies/organizations vs individuals, *P*=.001; physicians vs individuals, *P*=.03). Additionally, news agencies/organizations had higher PEMAT understandability scores than individuals (*P*=.01). Videos focused on disease knowledge scored higher in GQS (*P*<.001), PEMAT understandability (*P*<.001), and PEMAT actionability (*P*<.001) compared with videos covering surgical cases. PEMAT actionability scores were higher for outpatient cases compared with surgical cases (*P*<.001). Additionally, videos focused on surgical techniques had lower PEMAT actionability scores than those about disease knowledge (*P*=.04). The strongest correlations observed were between thumbs up and comments (r=0.92, *P*<.001), thumbs up and favorites (r=0.89, *P*<.001), thumbs up and shares (r=0.87, *P*<.001), comments and favorites (r=0.81, *P*<.001), comments and shares (r=0.87, *P*<.001), and favorites and shares (r=0.83, *P*<.001). Stepwise regression analysis identified “length (*P*<.001),” “content (*P*<.001),” and “physicians (*P*=.004)” as significant predictors of GQS. The final model (model 3) explained 50.1% of the variance in GQSs. The predictive equation for GQS is as follows: GQS = 3.230 − 0.294 × content − 0.274 × physicians + 0.005 × length. This model was statistically significant (*P*=.004) and showed no issues with multicollinearity or autocorrelation.

**Conclusions:**

Our study reveals that while most MR-related videos on TikTok were uploaded by certified physicians, ensuring professional and scientific content, the overall quality scores were suboptimal. Despite the educational value of these videos, the guidance provided was often insufficient. The predictive equation for GQS developed from our analysis offers valuable insights but should be applied with caution beyond the study context. It suggests that creators should focus on improving both the content and presentation of their videos to enhance the quality of health information shared on social media.

## Introduction

Mitral valve regurgitation (MR) is a condition characterized by the incomplete closure of the mitral valve, allowing blood to flow backward from the left ventricle into the left atrium during systole. MR is one of the most common heart valve diseases, affecting 2%-3% of the population, and its prevalence and severity increase with age. Among individuals older than 75 years, the prevalence of moderate or severe MR is over 10% [1,2]. The prognosis of MR depends on factors such as left ventricular function, etiology, severity, and duration of the disease.

MR is the second-most prevalent heart valve disease requiring surgical intervention in Europe [1]. In the United States, surveys indicate that nearly 2 million patients have moderate to severe MR, and this number is expected to increase to 4 million by 2030 [2]. In China, the detection rate of moderate and severe MR is 2.2% [3]. A large cross-sectional survey of a Chinese hospital population suggests that MR is the most common valvular heart disease in the country [4,5]. The proportion of patients with secondary MR gradually increases with age. A sampling survey showed MR rates of 18.4% among 35-year-old respondents and 25.2% among 65-year-old respondents. Additionally, secondary etiologies accounted for 51.7% of MR cases in 75-year-old respondents [5]. It is estimated that approximately 10 million patients with MR currently need treatment in China [6]. Despite the relative prevalence of MR, Chinese patients are often characterized by a high incidence of the disease, low levels of health awareness, and a low rate of surgical treatment. Fewer than 20% of patients have confirmed valvular disease before hospital admission, and only about 33% undergo invasive treatment [4]. Studies have shown that early diagnosis and intervention significantly improve survival rates [2,7-10]. Therefore, medical health education plays a crucial role in guiding people to recognize early symptoms, seek timely medical treatment, and gain access to early interventions.

Video-sharing media platforms, such as YouTube (Google LLC) and TikTok (ByteDance), have become integral to both personal and professional life. Videos featuring cartoons or documentary content are particularly popular, especially those related to occupational health and disease knowledge [11]. Many people access medical information online, and it is common for patients to look up information both before and after seeking medical treatment [12]. TikTok, a short-video–sharing social media platform that has gained significant popularity in recent years, offers a wide range of content across nearly all fields, including numerous health care–related videos. With up to 600 million active users in China, TikTok serves as a potentially crucial channel for distributing health information to consumers [13]. While TikTok holds great potential for disseminating public health information, the quality of disease-related videos on the platform varies widely. Researchers have evaluated the quality of TikTok videos related to cholelithiasis, COVID-19, diabetes, and chronic obstructive pulmonary disease [14-17]. However, the quality of videos about magnetic resonance (MR) imaging available on TikTok has not been assessed. Therefore, we investigated MR-related videos on TikTok to identify their upload sources, content, and features. We utilized quantitative scoring tools such as DISCERN, the Journal of the American Medical Association (JAMA) benchmark criteria, and the Global Quality Score (GQS) to conduct this evaluation. We also evaluated the educational guidance provided to the audience using the Patient Education Materials Assessment Tool for Audio-Visual Content (PEMAT-A/V) method.

## Methods

### Ethical Considerations

No clinical data, human specimens, or laboratory animals were involved in this study. Although our study is primarily based on a secondary analysis of mitral valve videos on TikTok, we placed significant emphasis on ethical considerations. We ensured that all research data were anonymized or deidentified to fully protect participant privacy. For any information that could potentially lead to personal identification, we implemented strict confidentiality measures, and access to the data was only provided to the research team.

### Retrieval Strategy

On September 9, 2023, from 09:00 AM to 11:00 AM, we conducted a search on the Chinese version of TikTok using the term “二尖瓣反流” (mitral regurgitation). To minimize research bias, we logged out of all personal accounts and refrained from using any filters. The first 100 retrieved videos were included in the study. We limited our analysis to the top 100 videos, as several studies [18,19] have confirmed that videos beyond this range do not significantly affect analysis outcomes. Videos featuring animals (n=2) and those consisting solely of images (n=12) were excluded from the study. For all included videos, the following features were recorded and analyzed: title, URL, uploader, uploader’s identity, website authentication, video duration (seconds), upload date, number of likes, number of comments, number of favorites, number of shares, and upload days (days since upload).

### Visual Classification

We classified video uploaders into 4 categories: doctors, individuals (eg, nonmedical professionals), news agencies (eg, online media, newspapers, television stations, and radio stations), and organizations (eg, hospitals, health authorities, research groups, universities, or colleges). The doctors were further classified as cardiac surgeons, cardiologists, or other health care professionals. The videos were categorized based on their content into the following types: disease knowledge, outpatient medical records, surgical cases, surgical techniques, and news/advertising.

### Video Content and Quality Assessment

The reliability of the videos was evaluated using the JAMA benchmark criteria and the modified DISCERN (mDISCERN) tool, while the quality of information was assessed using the GQS. Additionally, the educational impact of the video materials on the general public was measured using the PEMAT-A/V score. The quality assessment was conducted for all videos that met the eligibility criteria.

First, the JAMA benchmark criteria [20] were utilized to assess video reliability, encompassing 4 distinct criteria: (1) authorship; (2) attribution, including copyright information, references, and sources of content; (3) currency, including the initial date and subsequent updates; and (4) disclosure of conflicts of interest, funding, sponsorship, advertising support, and video ownership. Each criterion scores 1 point, with higher scores indicating greater reliability.

Second, the mDISCERN score, derived from the DISCERN tool, was used to analyze the reliability and quality of videos. It has been proven effective in assessing health-related video materials on other platforms, such as YouTube [21-24]. The mDISCERN score consists of 5 questions: (1) Are the aims of the video clear and achieved? (2) Are reliable sources of information used? (3) Is the information presented in a balanced and unbiased manner? (4) Are additional sources of information listed for patient reference? (5) Are areas of uncertainty mentioned? Each question is scored as 1 for “yes” and 0 for “no.” Higher scores indicate that the video is more reliable.

Third, we used the GQS to assess the quality of information in the videos. The GQS is widely recognized for evaluating the quality of health information on online video platforms [15,17,25]. It consists of 5 criteria: (1) poor quality (poor flow, most information missing, and not useful for patients); (2) generally poor quality (poor flow, some information provided but many important topics missing, of very limited use to patients); (3) moderate quality (suboptimal flow, some important information adequately discussed while others are poorly covered, somewhat useful for patients); (4) good quality (generally good flow, most relevant information listed, though some topics are not covered, useful for patients); and (5) excellent quality (excellent flow and very useful information for patients). Higher scores indicate higher-quality videos.

Finally, we used the PEMAT-A/V [26] to evaluate the educational impact of the video materials on the public. The PEMAT-A/V is specifically designed for assessing audiovisual materials and consists of 17 questions: 13 questions evaluate the understandability of the health information provided, while 4 questions assess the actionability of the recommendations. Responses are scored as “agree” (1), “disagree” (0), or “N/A.” The overall scores, as well as scores for the understandability and actionability sections, are calculated using the formula: “total points/total possible points × 100,” with higher scores indicating better understandability or actionability or both.

All authors are senior physicians with extensive clinical expertise in cardiology-related specialties. One person (NC) collected and downloaded all the videos. Two authors (NC and YC) evaluated the videos using the mDISCERN tool, JAMA score, GQS, and PEMAT-A/V. Scores were determined through discussion between them. In cases of disagreement, an arbitrator (XC) made the final decision. All authors subsequently approved each rating.

### Data Analysis

Data are expressed as means (SD) or medians (ranges). The Kruskal-Wallis test was used to compare data among multiple groups, while the Mann-Whitney *U* test was used for comparisons between 2 groups. The Dunnett multiple comparison test was applied for pairwise comparisons following a 2-way analysis of variance.

Because of the categorical nature of our data set and the nonnormal distribution of the data, we used the Spearman correlation coefficient to assess interparameter correlations. The correlation strength was categorized as follows: less than 0.25 indicates a poor relationship; 0.25–0.50 signifies a moderate relationship; 0.50–0.75 denotes a good relationship; and 0.75–1.00 represents an excellent relationship.

The Spearman correlation analysis revealed a significant (Figure 6) association between video parameters and the GQS. To assess the predictive capability of these video parameters for GQS, we used stepwise regression analysis, considering data type, characteristics, and other relevant factors. Initially, collinearity detection was performed to identify and remove variables with a variance inflation factor (VIF) greater than 5. Subsequently, GQS was used as the dependent variable, with uploaders, physicians, titles, content, duration, and length as independent variables in a stepwise approach. A *P* value of less than .05 was considered statistically significant.

We used the intraclass correlation coefficient (ICC) with a 2-way fixed-effects model to evaluate the scores between the reviewers (NNC and YLC). ICC values range from 0 to 1, where values less than 0.5 indicate poor consistency, 0.5-0.75 indicate moderate consistency, 0.75-0.90 indicate good consistency, and values greater than 0.90 indicate excellent consistency.

Statistical analysis was conducted using IBM SPSS version 19.0 software (IBM Corp.). Figures were created with GraphPad Prism version 9.5.1 (GraphPad Software) and OriginPro 2021 software (Origin Laboratories).

## Results

### Video Characteristics

In the final analysis, our study identified 88 eligible video files. The descriptive statistics for these videos are summarized in Tables 1 and 2, showing a total of 476,511 likes, 33,123 comments, 39,781 favorites, and 49,314 shares. The median video length was 255 seconds, with durations ranging from 4 to 1197 seconds, reflecting a wide spectrum of video lengths. Additionally, the median time since video upload was 61 (range 4-491) days, indicating the varied timing of content dissemination.

**Table 1:** General characteristics of the videos.

Characteristics	Mean (SD)	Median (range)
Video length (seconds)	336.71 (273.99)	255 (4-1197)
Duration on TikTok (days)	76.22 (68.87)	61 (4-491)
Thumbs up	5728.02 (23,785.95)	433 (23-206,000)
Thumbs up/day	20.07 (107.09)	2.22 (0.05-853.88)
Comments	396.20 (1506.29)	46 (1-13,000)
Comments/day	1.91 (6.69)	0.23 (0-53.89)
Favorites	479.29 (1382.07)	98 (0-9522)
Favorites/day	4.11 (15.03)	0.31 (0-107.51)
Sharing (counts)	581.64 (1607.70)	61 (0-12,000)
Sharing/day	2.76 (8.46)	0.55 (0-49.74)
JAMA^a^ score (0-4)	1.99 (0.11)	2 (1-2)
GQS^b^ score (0-5)	2.75 (0.79)	3 (1-5)
mDISCERN^c^ (0-5)	3.06 (0.23)	3 (3-4)
PEMAT^d^ understandability score, %	70.14 (17.74)	73.87 (16.67-100)
PEMAT actionability score, %	54.55 (45.26)	66.67 (0-100)

^a^JAMA: Journal of the American Medical Association.

^b^GQS: Global Quality Score.

^c^mDISCERN: modified DISCERN.

^d^PEMAT: Patient Education Materials Assessment Tool.

**Table 2:** Detailed characteristics of videos based on uploaders and content^a^.

Characteristic		n	Length (seconds)	Duration (days)	Thumbs up	Comments	Favorites	Sharing (counts)
**Uploaders**								
	Doctors	81	63 (4-491)	256.28 (4-1958)	421 (23-206,000)	46 (1-13,000)	92 (0-9522)	66 (0-12,000)
	Individuals	4	25.5 (874)	375.37 (74-949)	323 (140-1480)	38 (24-57)	48 (2-311)	7 (0-221)
	News agency/organizations	3	201 (55-277)	650.5 (490.2-724.6)	399 (255-1451)	87 (13-145)	77.5 (0-155)	343 (0-401)
	*P* value		.049	.17	.93	.94	.46	.22
**Doctors**								
	Cardiac surgeons	39	63 (5-491)	214 (8-1197)	766 (29-39,000)	94 (3-2733)	152 (0-2149)	165 (0-3206)
	Cardiologists	37	61 (4-210)	258 (4-1958)	270 (23-20,600)	32 (1-13,000)	50 (0-9522)	48 (1-12,000)
	Other health care professionals	5	79.4 (33.1)	337.8 (308.7)	349.6 (303.5)	43.4 (38.8)	67.4 (64.3)	57 (1-419)
	*P* value		.70	.81	.10	.17	.22	.36
**Professional titles**								
	Chief physician	58	63.5 (4-491)	255.5 (4-966)	545 (36-39,000)	65.5 (3-2733)	118.5 (0-6410)	86 (1-7263)
	Associate chief physician	14	56.86 (49.6)	314.9 (245.5)	103 (23-1416)	15 (1237)	18 (0-399)	8.5 (0-892)
	Attending/registered physician	9	113.67 (55.0)	705.33 (671.4)	300 (46-206,000)	54 (5-13,000)	20 (0-9522)	282 (1-12,000)
	*P* value		.03	.41	.04	.01	.008	.008
**Content**								
	Disease knowledge	50	66.5 (15-210)	73.1 (13.98)	699.5 (23-20,600)	79.5 (1-13,000)	164 (0-9522)	204.5 (0-12,000)
	Outpatient cases	19	66 (4-491)	64.74 (13.00)	226 (43-25,000)	16 (3-2695)	35 (1-2149)	19 (0-2007)
	Surgical cases	11	26.73 (19.43)	46.36 (15.60)	169 (29-9642)	22 (3-769)	20 (0-137)	12 (1-275)
	Surgical techniques	4	86.75 (38.95)	63.75 (35.80)	7740 (54-39,000)	482 (15-2733)	523 (24-1730)	693 (34-1783)
	News/advertising	4	126 (134.16)	64.75 (23.08)	303.5 (67-399)	50 (12-202)	5 (0-31)	8.5 (0-343)
	*P* value		.003	<.001	.03	.04	<.001	<.001

^a^Values are expressed as mean (SD) for continuous variables and median (range) for ordinal and discrete variables.

**Figure 1:**
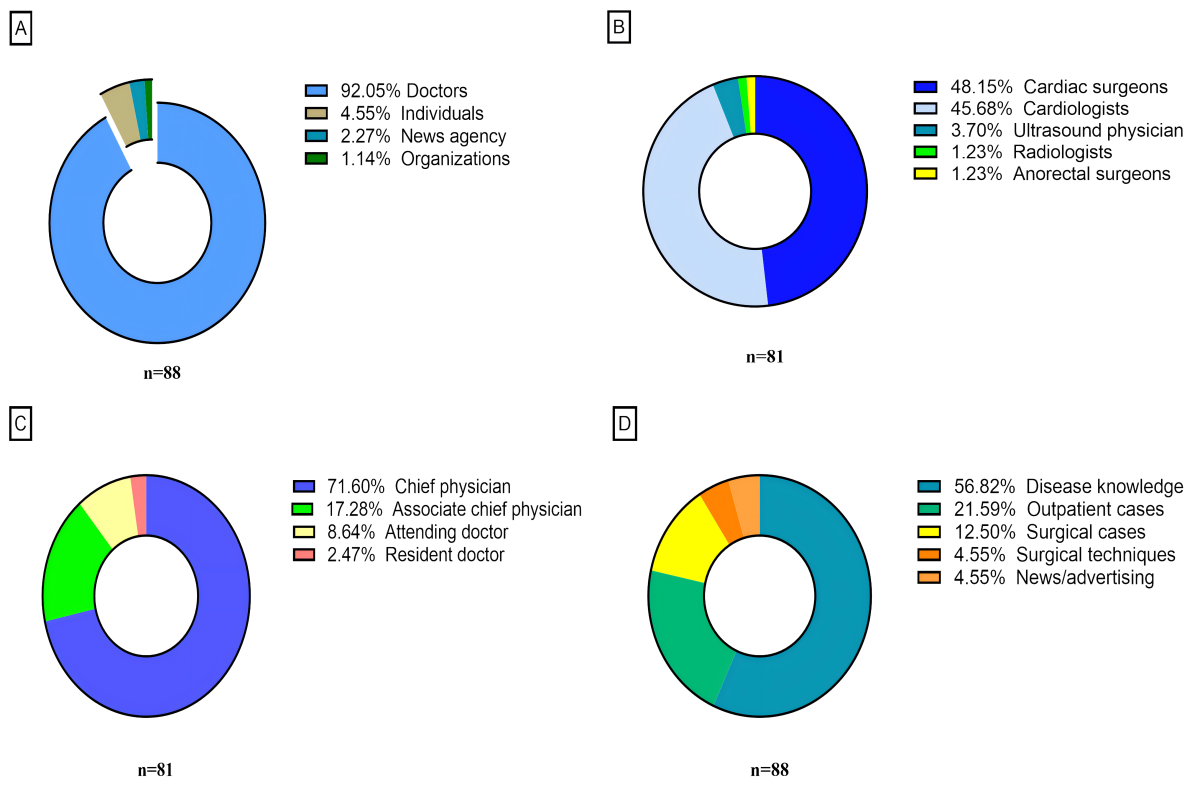
Percentage of videos on mitral valve regurgitation from different sources and with different contents on TikTok. (A) Video distribution based on uploaders. (B) Physician distribution. (C) Physicians’ professional and technical titles. (D) Video distribution based on content.

Initially, we obtained 100 videos. Of these, 84 were uploaded by physicians certified by TikTok, 13 by individual users, 2 by a news agency (online media), and 1 by an organization (hospital public account). After excluding videos that did not meet the inclusion criteria, 88 videos remained: 81 were uploaded by physicians, 4 by individual users, 2 by news agencies (online media), and 1 by an organization (hospital public account; Figure 1A). Among the physicians who uploaded videos, 39 were cardiac surgeons, 37 were cardiologists, 3 were sonographers, 1 was a radiologist, and 1 was an anorectal surgeon Figure 1B). Of the 81 videos uploaded by physicians, 58 were uploaded by chief physicians, 14 by associate chief physicians, 7 by attending physicians, and 2 by residents (Figure 1C). The videos were categorized by content (Figure 1D), with the most common topic being disease knowledge (n=50), followed by outpatient cases (n=19), surgical cases (n=11), surgical techniques (n=4), and news/advertising (n=4).

### Comparison of General Data

Table 2 provides a detailed account of the intergroup comparisons based on uploader category, physician type, academic title, and video content. In the uploader category, only video length showed a statistically significant difference, with videos from news agencies/organizations being significantly longer than those from other groups (*P*=.049). In terms of physician technical level, pairwise comparison analysis revealed significant differences in video length (chief physician vs associate chief physician, *P*=.02), number of likes (chief physician vs associate chief physician, *P*=.003), number of comments (chief physician vs associate chief physician, *P*=.01), number of favorites (chief physician vs associate chief physician, *P*=.006), and number of shares (chief physician vs associate chief physician, *P*=.009; attending/registered physician vs associate chief physician, *P*=.047). The number of upload days did not show any significant differences (*P*=.41). When classified by video content, statistical analysis revealed differences in all parameters among the groups (Table 2). Pairwise comparisons indicated significant differences in video duration (disease knowledge vs outpatient cases, *P*=.01), video length (disease knowledge vs outpatient cases, *P*=.001; outpatient cases vs surgical cases, *P*=.01), number of favorites (disease knowledge vs surgical cases, *P*=.007), and number of shares (disease knowledge vs outpatient cases, *P*=.006; disease knowledge vs surgical cases, *P*=.007). Because of the small sample size, pairwise comparisons for the number of likes and comments were not feasible. No significant statistical differences were observed in other classifications (Table 2).

### Video Quality and Reliability Assessments

We evaluated the videos using quantitative scoring tools such as mDISCERN, JAMA benchmark criteria, and GQS. Most videos (87/88) received a JAMA rating of 2, while 1 video received a rating of 1. Given that the JAMA criteria (range 0-4) were unable to effectively classify and assess video quality, they were excluded from the analyses of video quality and correlations.

The videos had a mean mDISCERN score of 3.06 (range 3-4) and a mean GQS of 2.75 (range 1-5), indicating that the TikTok videos exhibited fair quality and reliability. The PEMAT understandability score was 70.14 (range 16.67-100), and the PEMAT actionability score was 54.54 (range 0-100; Tables 1 and 3). This suggests that the TikTok videos were generally easy to understand but lacked effective implementation of recommendations, often providing either no suggestions or only general advice. The reviewers demonstrated good agreement on the mDISCERN scores (ICC 0.830, 95% CI 0.752–0.885), GQS scores (ICC 0.808, 95% CI 0.716–0.872), PEMAT understandability scores (ICC 0.966, 95% CI 0.946–0.978), and PEMAT actionability scores (ICC 0.829, 95% CI 0.748–0.885).

**Table 3:** The Global Quality Scores, mDISCERN^a^ scores, and PEMAT^b^ scores of the videos.

Scale, score			Values (N=88), n (%)
**mDISCERN**			
	1	0 (0)	
	2	0 (0)	
	3	83 (94)	
	4	5 (6)	
	5	0 (0)	
**Global Quality Score**			
	1	2 (2)	
	2	31 (35)	
	3	46 (52)	
	4	5 (6)	
	5	4 (5)	
**PEMAT—Understandability score**			
	0-60	26 (30)	
	61-80	32 (36)	
	81-100	30 (34)	
**PEMAT—Actionability score**			
	0	30 (34)	
	33.33-66.67	15 (17)	
	100	43 (49)	

^a^mDISCERN: modified DISCERN.

^b^PEMAT: Patient Education Materials Assessment Tool.

### Subgroup Analysis

**Figure 2:**
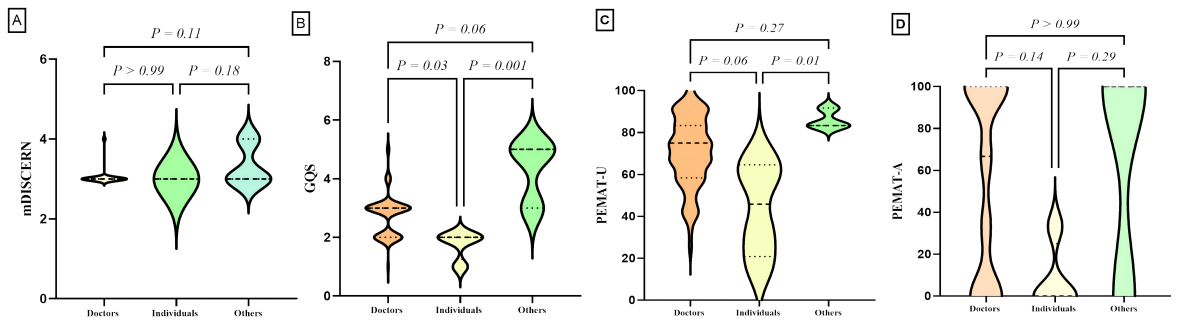
Quality assessment of videos based on uploaders. (A) Comparison of mDICERN scores among different uploaders. (B) Comparison of GQS between different uploaders. (C) Comparison of PEMAT-V scores among different uploaders. (D) Comparison of PEMAT-A scores among different uploaders. GQS: Global Quality Score; mDISCERN: modified DISCERN; PEMAT-A: Patient Education Materials Assessment Tool for Audio Content; PEMAT-V: Patient Education Materials Assessment Tool for Visual Content.

**Figure 3:**
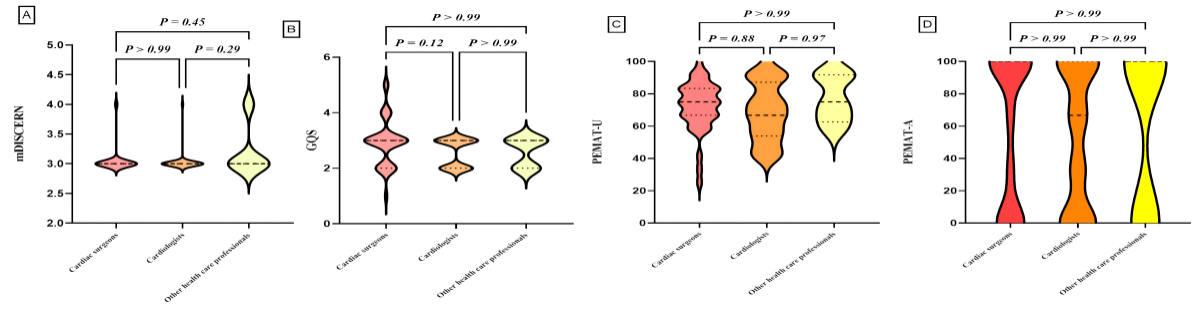
Quality assessment of videos based on doctors. (A) Comparison of mDICERN scores among different doctors. (B) Comparison of GQS between different doctors. (C) Comparison of PEMAT-V scores among different doctors. (D) Comparison of PEMAT-A scores among different doctors. GQS: Global Quality Score; mDISCERN: modified DISCERN; PEMAT-A: Patient Education Materials Assessment Tool for Audio Content; PEMAT-V: Patient Education Materials Assessment Tool for Visual Content.

**Figure 4:**
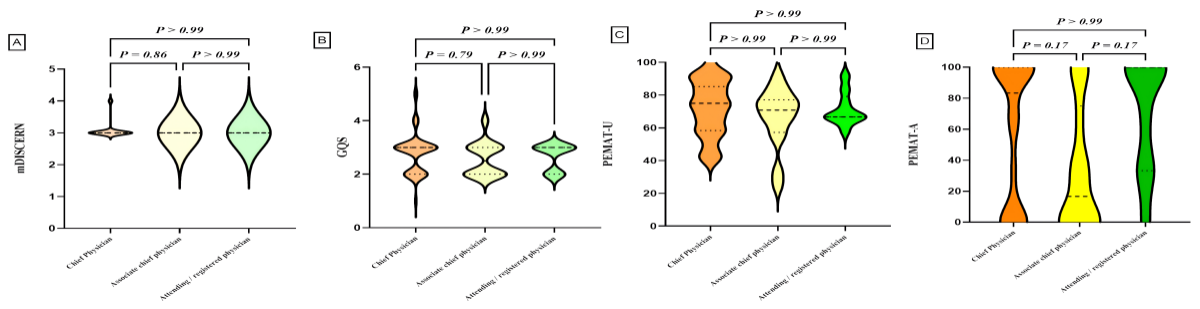
Quality assessment of videos based on professional titles. (A) Comparison of mDICERN scores among different professional titles. (B) Comparison of GQS between different professional titles. (C) Comparison of PEMAT-V scores among different professional titles. (D) Comparison of PEMAT-A scores among different professional titles. GQS: Global Quality Score; mDISCERN: modified DISCERN; PEMAT-A: Patient Education Materials Assessment Tool for Audio Content; PEMAT-V: Patient Education Materials Assessment Tool for Visual Content.

**Figure 5:**
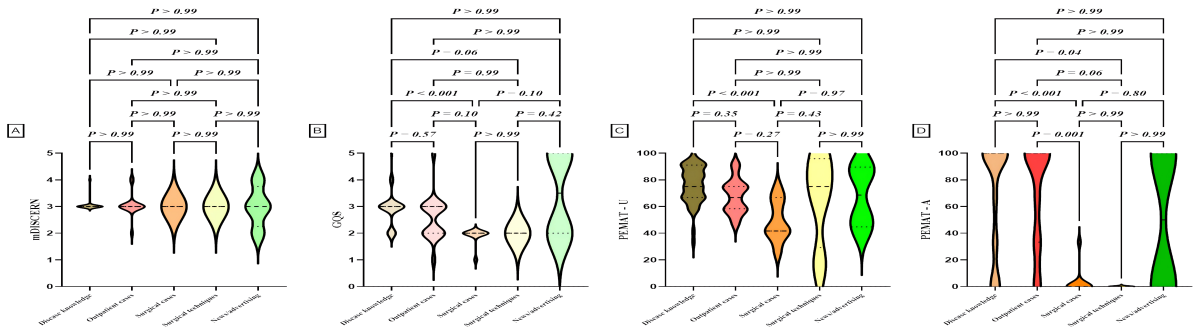
Spearman correlation analysis between video variables and the scores. GQS: Global Quality Score; mDISCERN: modified DISCERN; PEMAT-A: Patient Education Materials Assessment Tool for Audio Content; PEMAT-V: Patient Education Materials Assessment Tool for Visual Content.

We classified the videos based on uploaders, physician type, title, and content, and performed statistical analyses on the characteristic parameters of each video group. Subgroup comparisons are detailed in Table 4 and Figures 2-5. Differences were observed in GQS scores and PEMAT understandability scores among the subgroups. News agencies/organizations and doctors had higher GQS scores compared with individuals (news agencies/organizations vs individuals, *P*=.001; doctors vs individuals, *P*=.03). News agencies/organizations had higher PEMAT understandability scores compared with individuals (*P*=.01). No significant differences were found between subgroups based on physician type or title (Table 4). Analysis by video content classification revealed differences in GQS scores, PEMAT understandability scores, and PEMAT actionability scores. Videos focused on disease knowledge had higher GQS scores, PEMAT understandability scores, and PEMAT actionability scores compared with videos on surgical cases (*P*<.001 for all comparisons). Additionally, PEMAT actionability scores were higher for outpatient cases than for surgical cases (*P*<.001), and videos on surgical techniques had lower PEMAT actionability scores compared with videos on disease knowledge (*P*=.04).

**Table 4:** Quality assessment of videos based on uploaders and content^a^.

Characteristic		n	mDISCERN^b^	GQS^c^	PEMAT^d^ understandability score (%)	PEMAT actionability score (%)
**Uploaders**						
	Doctors	81	3 (3-4)	3 (1-5)	75 (25-100)	66.67 (0-100)
	Individuals	4	3 (3-3)	2 (1-2)	45.83 (16.67-66.67)	0 (0-33.33)
	News agency/organizations	3	3 (3-4)	5 (3-5)	83.33 (83.33-91.67)	100 (0-100)
	*P* value		.10	.002	.01	.12
**Doctors**						
	Cardiac surgeons	39	3 (3-4)	3 (1-5)	75 (25-100)	100 (0-100)
	Cardiologists	37	3 (3-4)	3 (2-3)	66.67 (41.67-100)	66.67 (0-100)
	Other health care professionals	5	3 (3-4)	3 (2-3)	75 (58.33-91.67)	100 (0-100)
	*P* value		.25	.11	.43	.77
**Professional titles**						
	Chief physician	58	3 (3-4)	3 (1-5)	75 (41.67)	83.34 (0-100)
	Associate chief physician	14	3 (3-3)	2.5 (2-4)	70.84 (25-91.67)	16.67 (0-100)
	Attending/resident doctor	9	3 (3-3)	3 (2-3)	66.67 (58.33-91.67)	100 (0-100)
	*P* value		.44	.52	.69	.01
**Content**						
	Disease knowledge	50	3 (3-4)	3 (2-5)	76.09 (13.98)	100 (0-100)
	Outpatient cases	19	3 (2-4)	3 (1-5)	67.82 (13.04)	70.18 (39.90)
	Surgical cases	11	3 (3-3)	2 (1-2)	49.24 (15.57)	0 (0-33.33)
	Surgical techniques	4	3 (3-3)	2 (2-2)	66.67 (36)	0 (0-0)
	News/advertising	4	3 (2-4)	3.5 (2-5)	67.63 (23.72)	0 (0-100)
	*P* value		.98	<.001	<.001	<.001

^a^Values are expressed as mean (SD) for continuous variables and median (range) for ordinal and discrete variables.

^b^mDISCERN: modified DISCERN.

^c^GQS: Global Quality Score.

^d^PEMAT: Patient Education Materials Assessment Tool.

### Correlation and Stepwise Regression Analysis

**Figure 6:**
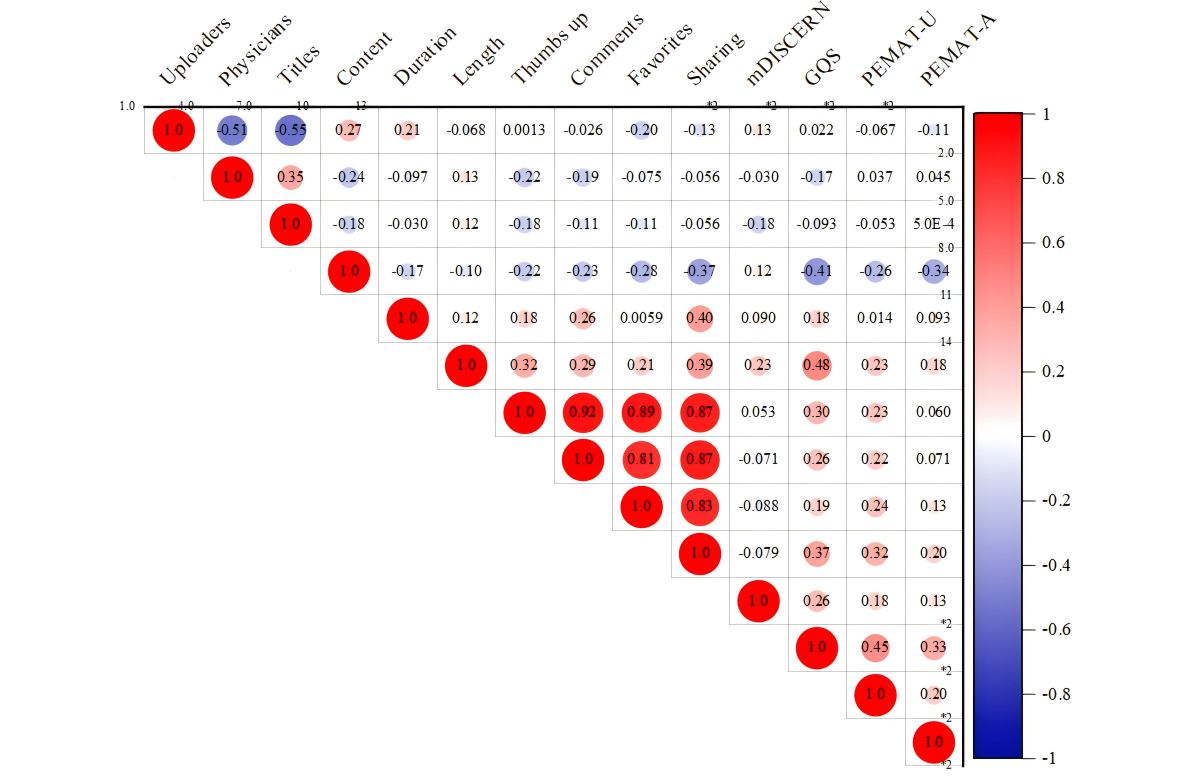
Quality assessment of videos based on contents. (A) Comparison of mDICERN scores among different contents. (B) Comparison of GQS between different contents. (C) Comparison of PEMAT-U scores among different contents. (D) Comparison of PEMAT-A scores among different contents. GQS: Global Quality Score; mDISCERN: modified DISCERN; PEMAT-A: Patient Education Materials Assessment Tool for Audio Content; PEMAT-V: Patient Education Materials Assessment Tool for Visual Content.

To explore correlations among various video parameters, we conducted a Spearman correlation analysis due to the presence of categorical variables and nonnormally distributed data (Figure 6). The analysis revealed strong correlations between video length, duration on TikTok, thumbs up, comments, favorites, and shares. Specifically, significant correlations were found between thumbs up and comments (*r*=0.92, *P*<.001), thumbs up and favorites (*r*=0.89, *P*<.001), thumbs up and shares (*r*=0.87, *P*<.001), comments and favorites (*r*=0.81, *P*<.001), comments and shares (*r*=0.87, *P*<.001), and favorites and shares (*r*=0.83, *P*<.001). However, the correlation between these video parameters and the evaluation tools is relatively weak (0.5>*r*>0.3). Specifically, the correlations are as follows: video length and GQS (*r*=0.48, *P*<.001), thumbs up and GQS (*r*=0.30, *P*<.001), and shares and GQS (*r*=0.37, *P*<.001). Additionally, we observed negative correlations between several video parameters: uploaders and physicians (*r*=–0.51, *P*<.01), uploaders and titles (*r*=–0.55, *P*<.01), physicians and content (*r*=–0.24, *P*<.05), content and comments (*r*=–0.23, *P*<.05), content and favorites (*r*=–0.28, *P*<.05), content and shares (*r*=–0.37, *P*<.01), content and GQS (*r*=–0.41, *P*<.01), content and PEMAT-Understandability (*r*=–0.26, *P*<.05), and content and PEMAT-Actionability (*r*=–0.34, *P*<.01).

The stepwise regression analysis produced 3 models, each incorporating additional predictors progressively (Tables 5 and 6). The final model (model 3), which included “length,” “content,” and “physicians” as predictors, demonstrated the strongest predictive power with an *R*^2^ of 0.501, indicating that these variables explain 50.1% of the variance in GQS. The adjusted *R*^2^ value was 0.482, and the model was statistically significant with an *F*_1,77_ ratio of 8.663 (*P*=.004). The Durbin-Watson statistic for model 3 was 1.960, indicating no significant autocorrelation in the residuals. The collinearity diagnostics revealed no multicollinearity issues, with tolerance values exceeding 0.9 and VIFs below 2.0 for all predictors in model 3. The predictive equation for GQS, based on model 3, is as follows: GQS = 3.230 − 0.294 × content – 0.274 × physicians + 0.005 × length.

**Table 5:** Stepwise regression analysis summary.

Model	Predictors included	*R* ^2^	Adjusted *R*^2^	SE of the estimate	*F* change (*df*)	*P* value	Durbin-Watson	ANOVA		
								*F* test (*df*)	*P* value	
1	Constant and length	0.285	0.276	0.59888	31.563 (1, 79)	<.001	2.194	31.563 (1, 79)	<.001	
2	Constant, length, and content	0.445	0.431	0.53112	22.444 (1, 78)	<.001	2.004	31.287 (1, 78)	<.001	
3	Constant, length, content, and physicians	0.501	0.482	0.50681	8.663 (1, 77)	.004	1.96	25.795 (1, 77)	<.001	

**Table 6:** Stepwise regression coefficients, statistical significance, and collinearity assessment.

Model and predictors			Unstandardized coefficients				Standardized coefficients (β)		*t* value (*df*)		*P* value		95% CI for B		Collinearity statistics		
			B		SE										Tolerance		VIF^a^
**1**																	
	Constant	2.241		0.103		N/A^b^		21.849 (79)		<.001		2.036 to 2.445		N/A		N/A	
	Length	0.006		0.001		0.534		5.618 (79)		<.001		0.004 to 0.008		1.000		1.000	
**2**																	
	Constant	2.779		0.146		N/A		19.087 (78)		<.001		2.489 to 3.069		N/A		N/A	
	Length	0.005		0.001		0.464		5.412 (78)		<.001		0.003 to 0.007		0.970		1.031	
	Content	–0.286		0.060		–0.406		–4.738 (78)		<.001		–0.407 to –0.166		0.970		1.031	
**3**																	
	Constant	3.230		0.207		N/A		15.618 (77)		<.001		2.818 to 3.642		N/A		N/A	
	Length	0.005		0.001		0.457		5.586 (77)		<.001		0.003 to 0.006		0.969		1.032	
	Content	–0.294		0.058		–0.416		–5.089 (77)		<.001		–0.409 to –0.179		0.968		1.033	
	Physicians	–0.274		0.093		–0.237		–2.943 (77)		.004		–0.459 to –0.089		0.998		1.002	

^a^VIF: variance inflation factor.

^b^N/A: not applicable.

## Discussion

### Principal Findings

This cross-sectional study revealed that most MR-related videos on TikTok were uploaded by certified physicians, predominantly cardiac surgeons or cardiologists. Notably, all physician identities were verified by TikTok to ensure professionalism and scientific accuracy. The video content quality, assessed using the mDISCERN tool, yielded satisfactory scores; however, the GQS scores did not meet the expected standards. The evaluation of the educational impact of the videos showed that a significant majority achieved PEMAT understandability scores above the threshold, indicating high levels of comprehension and acceptance among viewers. However, there was considerable variation in the PEMAT actionability scores, with approximately half of the videos falling below satisfactory levels. This suggests that many videos have limited practical applicability. Our analyses yielded several noteworthy observations.

### Mitral Regurgitation Video Characteristics and Audience Interaction Analysis

Our study found that the 88 videos received a total of 476,511 thumbs up, 33,123 comments, 39,781 favorites, and 49,314 shares. This high level of viewer engagement highlights significant interest in MR content and a notable degree of interactivity, which contrasts with findings from previous studies [27,28]. Most videos were uploaded by cardiologists certified by TikTok, primarily cardiac surgeons and cardiologists, with some contributions from the ultrasound department. This indicates a strong willingness among medical professionals to share their expertise on the TikTok platform. In addition to videos uploaded by doctors, there were contributions from news media, nonprofit medical official accounts, and individuals. The content spanned various topics, including disease knowledge, outpatient cases, surgical cases, surgical techniques, and personal experiences. This diversity addresses the needs of different audiences for medical information and has a positive impact on public health awareness.

Furthermore, our results suggest that video length is a significant distinguishing factor, consistent with previous findings [12]. Analysis by different classification criteria revealed statistical differences among groups (Table 2). Specifically, videos uploaded by chief physicians and those focused on disease knowledge were notably longer and received more thumbs up, comments, favorites, and shares. A detailed examination of the videos revealed that the discrepancy may be attributed to the public’s greater trust in physicians with higher technical ranks and their interest in understanding disease knowledge, often seeking assistance through consultation. Notably, 1 video by an attending physician achieved exceptionally high engagement, with 206,000 thumbs up, 13,000 comments, and 12,000 shares. This video stood out because the physician used colloquial language to explain disease knowledge in an accessible and sincere manner, offering valuable insights for content creators on how to produce higher-quality videos.

### Video Ratings and Quality Evaluation

Our study used various assessment tools to evaluate video quality. First, we used the modified DISCERN tool to gauge the reliability of the videos. The mDISCERN results indicated that the videos had moderate credibility—acceptable but not entirely satisfactory. Subgroup analysis did not reveal significant differences among the groups. Upon closer examination, we observed that video creators often neglected to provide sources of information, and TikTok’s lack of a mandatory review mechanism meant that the question regarding additional sources for patient reference (Are additional sources of information listed for patient reference?”) was not scored. This highlights the importance for content creators to include clear and reliable sources to enhance the credibility of their content.

Second, we assessed the overall quality of the video content using the GQS. The average GQS score was 2.75 (range 0-5), which is near the threshold for moderate quality. This finding aligns with Collà Ruvolo et al [28] but is notably lower than the results reported by Morra S et al [27]. Subgroup analysis revealed that videos from doctors and news agencies/organizations had higher GQS scores compared with those from individuals (Figure 2B). Additionally, videos focusing on disease knowledge received higher GQS scores than those covering surgical cases (Figure 5B).

Lastly, we used the PEMAT-A/V tool to assess the educational significance of the videos. The PEMAT-A/V results showed that the understandability score was 70.14%, while the actionability score was 54.54%. According to Shoemaker et al [26], a PEMAT score exceeding 70% indicates that the content is sufficiently understandable and actionable. Our findings suggest that while the videos are generally understandable, they fall short in terms of actionability. This observation is consistent with the findings of Kanner et al [29], who reported similar issues with TikTok and YouTube videos on overactive bladder. Subgroup analysis revealed that videos from media organizations scored higher on the PEMAT-A/V understandability and actionability compared with those from individuals (Figure 2C). Additionally, videos focused on disease knowledge had higher PEMAT-A/V understandability scores compared with those on surgical cases. Both disease knowledge and outpatient cases achieved higher scores than surgical cases in terms of understandability and actionability.

Integrating these assessment outcomes, we found that videos produced by doctors or news agencies/organizations with a medical background, particularly those focusing on disease knowledge, tend to be of higher quality compared with others. Patients interested in MR or similar topics are advised to prioritize watching these videos for their informative and reliable content.

### Correlation and Stepwise Regression Analysis Between Video Quality and Video Characteristics

We conducted an in-depth analysis of MR video content on TikTok. Using Spearman correlation analysis, we assessed the relationship between video characteristics and quality metrics. Additionally, we used stepwise regression analysis to evaluate the predictive capacity of video parameters on quality scores—an innovative approach not previously explored in similar studies. Our correlation and stepwise regression analyses have provided a comprehensive view of the factors influencing video quality in the context of MR content on TikTok. However, interpreting our results, especially the observed negative correlations, should be done with an understanding of the statistical methods used.

The strong positive correlations observed among video length, duration, and engagement metrics—such as thumbs up, comments, favorites, and shares—highlight a significant interrelationship between these variables. This finding supports existing literature, which suggests that content with higher audience engagement is more likely to be widely disseminated, a concept explored by Weng et al [30] in the context of social media network effects. The weaker correlation between video characteristics and GQS scores suggests that viewer engagement, while significant, does not necessarily reflect the intrinsic quality of the content. This observation aligns with Berger and Milkman [31], who found that emotional appeal and content novelty are key drivers of engagement, regardless of the content’s educational or informational value. Additionally, the negative correlations between certain video parameters, such as the uploader’s professional role and content type, may stem from the statistical methodology used rather than indicating a true inverse relationship.

The stepwise regression analysis highlighted “length,” “content,” and “physicians” as significant predictors of GQS scores, with the final model demonstrating substantial predictive strength. The model suggests that longer videos and those featuring physicians are associated with higher GQS scores, while certain content types may be linked to lower scores. However, the negative coefficients for “content” and “physicians” should be interpreted with caution, as they may not directly indicate causal relationships.

### Practical Significance

Mild MR often goes unnoticed and is frequently detected through ultrasound scans. In the digital age, individuals increasingly rely on video platforms to seek health information and share experiences. The Chinese government acknowledges the value of these platforms in health education and encourages medical professionals to actively disseminate health science knowledge [32]. This initiative is especially pertinent on TikTok, a leading short-video platform in China that is brimming with disease-related content. Our study reveals that cardiologists’ contributions to TikTok offer professional and scientifically sound content on MR, providing valuable guidance to patients and potentially reducing unnecessary medical consultations. However, challenges persist, including limitations in TikTok’s recommendation algorithms and constraints on video length, which may restrict the depth of information conveyed. To establish a reliable source of high-quality medical content, it is crucial for TikTok to enhance its certification processes for content creators and implement robust content review mechanisms. These measures will ensure that viewers receive accurate and comprehensive health information that meets their needs and supports informed decision-making.

### Strengths and Limitations

Our study provides robust insights into health information dissemination on TikTok, highlighting the importance of content validation by certified medical professionals to ensure the reliability of health messages. By comprehensively measuring audience engagement through metrics such as likes, comments, favorites, and shares, we gain a multidimensional understanding of video impact. Additionally, the innovative use of stepwise regression analysis to predict video quality based on specific characteristics introduces a novel approach to digital health communication. The methodological rigor, demonstrated through the use of Spearman correlation and regression models, strengthens the study’s findings. Additionally, the focus on practical implications provides valuable guidance for both content creators and viewers.

Despite these strengths, the study has several limitations. One limitation is the potential bias in the statistical methodology, which may affect the interpretation of negative correlations. The content analyzed might not fully represent the entire range of MR topics, potentially limiting the applicability of the findings. Additionally, the study does not account for the influence of TikTok’s recommendation algorithms, which could significantly impact video visibility and engagement levels. The diverse demographics of TikTok users and their varying interactions with health content are not fully accounted for, which could impact the study’s conclusions. As a correlational study, it does not infer causality, and the findings may not be generalizable to other platforms or contexts. Furthermore, the study’s scope is limited to a specific period, which may not capture the dynamic nature of content evolution and user engagement on social media platforms over time. This temporal limitation may affect the long-term applicability and stability of the findings.

### Conclusions

Currently, most MR-related videos on TikTok are uploaded by certified physicians, which ensures their professional and scientific reliability. However, the overall video-quality scores were suboptimal. While many videos provided educational value, the guidance offered was insufficient, and video production quality needs improvement. Notably, video prevalence was only weakly correlated with quality. The predictive equation for GQS developed from our analysis provides valuable insights but should be applied cautiously beyond the context of this study. This suggests that creators should focus on enhancing both the content and presentation of their videos to improve the quality of health information shared on social media. Additionally, TikTok should bolster its review mechanisms to elevate the standard of medical content, thereby benefiting users and strengthening the integrity of online health communication.

## Abbreviations

GQS: Global Quality Score
ICC: intraclass correlation coefficient
JAMA: The Journal of the American Medical Association
mDISCERN: modified DISCERN
MR: mitral valve regurgitation
PEMAT-A/V: Patient Education Materials Assessment Tool for Audio-Visual Content
VIF: variance inflation factor

## Data Availability

The data for this study, derived from the TikTok platform, have been anonymized to protect privacy, with URLs and titles retained for research integrity. Because of privacy concerns, these data are not publicly accessible. However, we are committed to sharing the data upon request for legitimate research purposes, in accordance with privacy protection principles and data sharing policies. Interested researchers should contact the corresponding author for access.
